# Evaluating the feasibility and effectiveness of a critical care discharge information pack for patients and their families: a pilot cluster randomised controlled trial

**DOI:** 10.1136/bmjopen-2014-006852

**Published:** 2015-11-27

**Authors:** Suzanne Bench, Tina Day, Karina Heelas, Philip Hopkins, Catherine White, Peter Griffiths

**Affiliations:** 1 Florence Nightingale Faculty of Nursing and Midwifery, King's College, London, UK; 2 Florence Nightingale Faculty of Nursing and Midwifery, King's College, London, UK; 3 Critical Care Unit, King's College Hospital NHS Foundation Trust, London, UK; 4 ICU Steps Charity, Milton Keynes, UK; 5 Faculty of Health Sciences, University of Southampton, Southampton, UK

## Abstract

**Objectives:**

To evaluate the feasibility and effectiveness of an information pack, based on self-regulation theory, designed to support patients and their families immediately before, during and after discharge from an intensive care unit (ICU).

**Design and setting:**

Prospective assessor-blinded pilot cluster randomised controlled trial (RCT; in conjunction with a questionnaire survey of trial participants’ experience) in 2 ICUs in England.

**Participants:**

Patients (+/− a family member) who had spent at least 72 h in an ICU, declared medically fit for discharge to a general ward.

**Randomisation:**

Cluster randomisation (by day of discharge decision) was used to allocate participants to 1 of 3 study groups.

**Intervention:**

A user-centred critical care discharge information pack (UCCDIP) containing 2 booklets; 1 for the patient (which included a personalised discharge summary) and 1 for the family, given prior to discharge to the ward.

**Primary outcome:**

Psychological well-being measured using Hospital Anxiety and Depression Scores (HADS), assessed at 5±1 days postunit discharge and 28 days/hospital discharge. Statistical significance (p≤0.05) was determined using χ^2^ and Kruskal-Wallis (H).

**Results:**

158 patients were allocated to: intervention (UCCDIP; n=51), control 1: ad hoc verbal information (n=59), control 2: booklet published by ICUsteps (n=48). There were no statistically significant differences in the primary outcome. The a priori enrolment goal was not reached and attrition was high. Using HADS as a primary outcome measure, an estimated sample size of 286 is required to power a definitive trial.

**Conclusions:**

Findings from this pilot RCT provide important preliminary data regarding the circumstances under which an intervention based on the principles of UCCDIP could be effective, and the sample size required to demonstrate this.

**Trial registration number:**

Current Controlled Trials ISRCTN47262088; results.

**Strengths and limitations of this study**

- This is one of few randomised controlled trials that have evaluated critical care discharge information resources and the first to evaluate the use of an intervention, which includes a personalised patient discharge summary.
- Results suggest that information based on self-regulation theory is feasible to deliver, may improve patients’ understanding of their critical illness and may help optimise critical illness rehabilitation.
- The a priori enrolment goal was not reached and attrition was high.
- The study had insufficient statistical power to determine any outcome benefit.

## Introduction and background

Providing information is an important element of effective critical illness rehabilitation care,^1^ ^2^ yet at the time of discharge from an intensive care unit (ICU) to a general care environment (ward), some patients and relatives report not receiving any information^3^ ^4^ or receiving ad hoc verbal information, sometimes accompanied by a leaflet or booklet.^5^

Patient-focused healthcare provision, which promotes shared decision-making, is widely advocated.^6–9^ Guidelines from the Department of Health in England (p.16) recommend that acutely ill patients should be “encouraged to actively participate in decisions related to their recovery…”^10^; this, however, requires the provision of appropriate information. To be effective, ICU discharge information needs to take account of the cognitive problems and fatigue apparent in many patients recovering from critical illness.^11^ Any written information must also acknowledge the heightened anxiety experienced by both patients and relatives at this time^11^ and reflect the differing information needs of both groups at various time points.^4^ ^5^ Our intervention was designed to address all of these elements, in contrast to the interventions described in the few studies which have previously evaluated written ICU discharge information resources.^12–15^

There is currently little evidence to support best practice with regard to ICU discharge information delivery.^5^ Data from the few studies, which have evaluated written resources, suggest that it can improve family members’ knowledge and satisfaction^13^ ^14^ and reduce their anxiety^15^ during and after ICU discharge. The results of a multicentre UK randomised controlled trial (RCT) also suggest that written information may help lower patients’ levels of depression and symptoms of posttraumatic stress disorder (PTSD), when provided as part of a broader rehabilitation strategy.^12^ These limited data justify further investigation of the key elements of ICU discharge information that lead to positive health outcomes.

## Objectives

This paper reports a RCT designed to (1) provide an initial evaluation of a user-centred critical care discharge information pack (UCCDIP), (2) inform decisions regarding its further development and evaluation, and (3) estimate the sample size required to power a definitive trial.

## Methods

### Design

**Figure 1:**
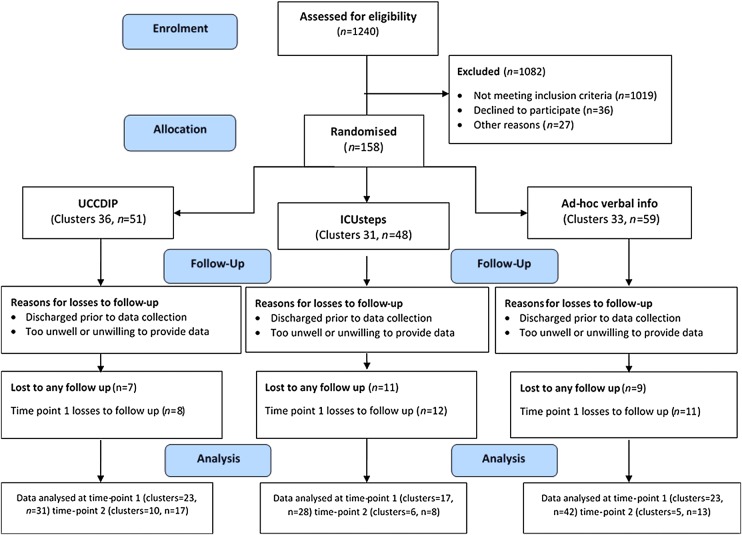
Flow of participants (ICU, intensive care unit; UCCDIP, user-centred critical care discharge information pack).

We designed an external pilot pragmatic RCT (figure 1) to provide initial data regarding the feasibility and effectiveness of UCCDIP. In accordance with the definition of an external pilot,^16^ an assessment of the primary outcome was included. To reduce the chance of between-group contamination, the design also incorporated cluster randomisation, where groups of participants (as opposed to individuals) were allocated to study arms. During the trial, a questionnaire survey was conducted to determine the experiences of trial participants and nursing staff.

In line with best practice,^17^ ^18^ a former patient and Trustee of ICUsteps (an ICU patient and relative support charity) was included on the project team. Recruitment took place between 8 August 2011 and 4 May 2012, and informed patient consent was obtained prior to data collection. The trial was registered on The International Standard Randomized Controlled Trial Number (ISRCTN) database (ISRCTN47262088) 5 months after recruitment of the first patient. This delay was due to an administrative problem between trial registry and the funding body. The full trial protocol is available in online supplementary file 1.

### Setting and participants

The study took place in two ICUs (medical n=14 beds and surgical n=18 beds) within a single teaching hospital in central London, England; providing care for a mixed medical, surgical and trauma patient population, requiring level 2 (high dependency) or level 3 (intensive) care.^19^ Both units functioned as one department; staff rotated between units and patients were allocated to a bed based on availability; regardless of whether they required medical or surgical care. Patients over 18 years were considered for inclusion into a cluster if they had spent at least 72 h in the ICU (table 1). The intention was to recruit all eligible patients, declared medically fit for discharge to a general ward Monday to Friday (08:00–20:00) and a nominated relative. Inclusion criteria were based on best practice guidelines surrounding ICU discharge,^1^ with an aim to avoid including overnight stay elective surgical patients whose discharge had been delayed due to the unavailability of a ward bed.

**Table 1:** Inclusion/exclusion criteria for individual participants

Inclusion criteria	Adult patients (>18 years) Adult family members of eligible patients (>18 years) Elective or emergency admissions in the ICU ≥72 h Patients identified for discharge to a general ward setting within the hospital Elective discharges between 08:00 and 20:00 Monday to Friday
Exclusion criteria	Patients for whom active treatment had been withdrawn Inability to verbally communicate in or read English Involvement in a phase I focus group study^4^

ICU, intensive care unit.

All participants (patients and relatives) were recruited while the patient was in ICU. Potential patient participants were consented the day prior to a formal discharge decision wherever possible. For patients unable to provide informed written consent at the point of ICU discharge, personal consultee declarations,^20^ usually from the patient's next of kin, were sought. Informed consent from the patient was then obtained prior to data collection on the ward. The relatives of all recruited patients were given study information when they visited the ICU, or telephoned and invited to participate. Written consent was obtained from relatives who agreed to participate during their next hospital visit. All participants were allocated a trial number. All members of a family unit were given the same number, prefixed by either a P or R (eg, P1 for the patient and R1 for the relative). The assigned trial number was used across all data collection forms, enabling anonymised data from all sources to be matched and comparisons made between patient participants (and their relatives), their characteristics and the outcome data.

### Intervention

Drawing on self-regulation theory (SRT),^21^ ^22^ our intervention (UCCDIP) was developed using data from a previous focus group study.^4^ UCCDIP consists of two booklets, one for the patient and one for the relatives (see online supplementary file 2). The front page of the patient booklet includes an individualised patient discharge summary, written by ICU bedside nurses; trained to use a template designed by the project team (CW, PH). The pack also contains information aimed at preparing the patient/family for ICU discharge and the transition to the ward. It encourages active participation by offering space for expression of individual questions and concerns. It also includes diary pages for both the patient and family to record their thoughts and feelings during the in-hospital recovery period, if they wish. In accordance with SRT, UCCDIP was designed as an information resource, to help users develop revised illness perceptions, more consistent with effective coping.^22^ ^23^ The intervention is further described in Bench *et al*.^24^

Participants in all three study arms received usual care, which consisted ‘ad hoc’ verbal ICU discharge information provided by a variety of healthcare professionals. No guidance was given for this and the quality and quantity of information delivered was totally dependent on each staff member's usual practice. To minimise the risks of additional attention, given to participants in the intervention group having a placebo effect,^25^ ^26^ in addition to the ‘ad hoc’ information given to all participants, a second ‘attention control’ group received alternative written information in the form of a booklet produced by the ICUsteps charity.^27^ In contrast to UCCDIP, the information in the ICUsteps booklet covered the whole trajectory of critical illness from ICU admission to after hospital discharge. In addition, the ICUsteps booklet did not offer opportunities for participants to reflect on their experience or feelings, or prompt them to consider their individual information needs.

#### Intervention delivery

Immediately after recruitment, patient participants in all clusters were given an identical looking folder containing a covering letter, study information and, where applicable, written discharge information (either UCCDIP or the ICUsteps booklet). These folders accompanied patients when they were discharged to the ward.

For participants randomised to a cluster receiving the intervention, the bedside ICU nurse orientated the patient to the contents of the UCCDIP and the research nurse (KH) checked that the patient discharge summary was completed according to agreed guidelines.^24^ The bedside nurse did not go through the written booklet given to participants in the cluster allocated to receive the ICUsteps booklet.

Recruited relatives were allocated to the same study group as the patient, but it was left up to the patient to pass the information on to their family. Although UCCDIP contained an information book specifically for relatives, they were not included in the discussion between the bedside nurse and the patient, unless they happened to be present on the unit at the time.

### Outcomes

The primary outcome was individual patients’ sense of psychological well-being (specifically anxiety and depression), measured using the internationally validated Hospital Anxiety and Depression Score (HADS) tool^28^ with a threshold ≥8 used to identify possible clinical cases of anxiety and/or depression.^29^ Secondary outcomes included individual patients’ perceptions of coping, measured using the Brief Coping Orientations to Problems Experienced (BCOPE) tool^30^ and relatives’ sense of psychological well-being (anxiety, depression and coping assessed using HADS and BCOPE). In addition, patients’ perceptions of their ability for self-care were measured using the Patient Enablement Instrument (PEI).^31^ A locally designed questionnaire survey described the discharge experiences of all recruited patients and their relatives. The views of the ICU and ward nursing staff about UCCDIP were also explored using the questionnaire survey and have been previously published.^32^ Face and content validity of the questionnaire were reviewed by the patient advisory group, but no pilot was undertaken prior to its use. Table 2 details the instruments and measures used to assess both primary and secondary outcomes.

**Table 2:** Data collection instruments and measures

Participant	Outcome	Instrument	Measurement
Patient	Anxiety and depression	HADS	On ward, 5 (±1) days post-ICU discharge Hospital discharge or 28 days
Patient	Perceptions of coping	BCOPE	On ward, 5 (±1) days post-ICU discharge Hospital discharge or 28 days
Patient	Perceptions of self-care ability	PEI	On ward, 5 (±1) days post-ICU discharge Hospital discharge or 28 days
Patient	Discharge experience	Questionnaire	Prior to hospital discharge
Relative	Anxiety and depression	HADS	On ward, 5 (±1) days postpatient's ICU discharge Patients’ hospital discharge or 28 days
Relative	Perceptions of coping	BCOPE	On ward, 5 (±1) days postpatient's ICU discharge Patients’ hospital discharge or 28 days
Relative	Discharge experience	Questionnaire	Prior to patient's hospital discharge
ICU and ward nurses	Views about UCCDIP	Questionnaire	End of trial period

BCOPE, Brief Coping Orientations to Problems Experienced; HADS, Hospital Anxiety and Depression Scale; ICU, intensive care unit; PEI, Patient Enablement Instrument; UCCDIP, user-centred critical care discharge information pack.

To assess the effects of the intervention on early in-hospital psychological well-being, outcome data from patient and relative participants were collected on the ward on two occasions after ICU discharge: 1 week (defined as 5±1 day) and at hospital discharge or 28 days, whichever was sooner. The questionnaire survey was completed prior to a patient's hospital discharge (participants) or at the end of the trial period (nurses) (table 2).

Data were collected by one researcher (SB), with back-up provided (TD). To maximise the chance of retrieving a full data set, researchers facilitated completion of forms (by reading questions and writing responses) for some of the less able patients. Relatives were asked to complete forms on the ward or at home, and to return them directly to the research team (by email or post) or to leave them for collection at the patient's bedside.

Demographic information, length of ICU stay, ICU readmissions, medical history, Acute Physiology and Chronic Health Evaluation (APACHE) II scores,^33^ therapies received and complications pertinent to the critical illness period were retrieved from local databases and medical notes immediately after each patient participant was recruited. At this point, participating relatives were also asked to complete a form detailing their demographics, previous experiences of critical illness and relevant medical history (such as anxiety and/or depression). The number of unit discharges per day during the trial period and other feasibility data, such as comments received from staff and challenges associated with intervention delivery, were also recorded by the research nurse. All completed discharge summaries were photocopied and retained.

### Sample

Based on data from a previous RCT by Gammon and Mulholland,^34^ which examined the effect of information giving on the HADS of a sample of perioperative patients, the sample size calculation for the present trial was carried out using G*Power V.3.1.2. To detect a moderate effect size of 0.6 (mean difference of 3 units, SD 5) with a power of 80% and α set at 0.05, a minimum of 45 participants in each group were required. To account for attrition and the likely variation in ICU discharges in each cluster (discharge day), our accrual target was 50 participants in each of three study arms.

Sample size was not based on an intracluster correlation (ICC) calculation, as insufficient information was available to determine the real extent of any homogeneity between clusters. As only those discharged on weekdays were recruited, it was anticipated that every day would produce a fairly similar and randomly determined clinical case load (local data 2010/2011), thus limiting the likelihood of homogeneity (eg, similar diagnoses) within and heterogeneity (eg, care led by different medical teams) between clusters. The intervention for each cluster was also preallocated on a random basis, thus minimising (although not eradicating) the chance of clusters being homogeneous.

There was no opportunity to influence cluster size, and so to maximise the chance of recruiting the required number of participants, data collection was planned to continue for at least 132 days, providing 44 potential clusters in each of the three study arms, significantly greater than the minimum of five recommended by the UK Medical Research Council.^35^

### Randomisation

As described by Hayes and Moulton,^36^ this trial used cluster randomisation for pragmatic reasons, with an aim to reduce the risk of cross-contamination between study arms. All patient participants (and their relative where applicable) discharged from either ICU on a particular day (a cluster) were allocated to one of the three study groups (figure 1). Particular days of the week were not allocated to specific arms; instead each day was randomly allocated to a study arm and treated as a distinct cluster, based on the allocation schedule.

To ensure that the sequence of allocation was not predictable, the day on which the intervention, control and attention control was used was randomly assigned using a computer-generated random sequence, prepared by a statistician. This involved simple randomisation with no blocking or stratification for defined variables.

The allocation schedule was prepared by persons independent of the trial and concealed by being wrapped in a blank piece of paper and placed inside sequentially numbered, sealed envelopes. These envelopes were signed across the seal and opened by the research nurse (KH), in the presence of another member of the research team, only after a recruited patient was identified for discharge to the ward on a particular day. The clinical ICU staff only became aware of which study group the patient was allocated to when the bedside nurse was provided with a study pack to give to the patient. Allocation concealment for the whole cluster was revealed after the first envelope was opened on any day.

### Blinding

It was not possible to achieve full blinding during this trial as intervention delivery required input by healthcare staff and trial participants. Those collecting and analysing data were, however, blinded by the use of codes, which were not broken until after data analysis. Blinding was compromised if participants revealed their information pack to the data collector. In most instances, however, as all participants received a folder, identical on the outside, data collectors (SB/TD) remained blinded to the allocation.

### Statistical methods

Data analysis was based on an ‘intention-to-treat’ strategy^37^ and statistical significance was set at p≤0.05. Average values for sample characteristics, HADS, BCOPE, PEI scores and questionnaire responses in each of the study groups were compared using χ^2^ for categorical data and Kruskal-Wallis (H) for data of at least ordinal level. In addition, Friedman's test (χ^2^r) was used to explore associations between the different types of coping. Difference in HADS (the primary outcome) between the three study groups was also tested using the Statistical Analysis System (SAS) V.9.3 (SAS Institute Inc, Cary, North Carolina, USA), Generalised Mixed Models procedure (GLIMMIX) that adjusted for clustering (by fitting a random intercepts model) and recruitment weekday. At time point 2, it was not possible to fit random intercepts because the G-side matrix was always not positive definite. We followed the CONSORT guidance and did not conduct baseline statistical comparisons between study groups.^38^

This paper reports outcome data from the patient participants only, with reference to the demographics and attrition data collected from the sample of relatives.

## Results

Two hundred and twenty-one (18%) of the 1240 screened patients met the inclusion criteria and 158 of these were recruited in 100 clusters, each containing 1–5 patients (table 3). The distribution by cluster size was as follows: one patient (n=66), two patients (n=21), three patients (n=6), four patients (n=3) and five patients (n=4). Fifty-one (32%) patient participants were allocated to the intervention group (UCCDIP), 59 (37%) to control group 1 (ad hoc verbal information) and 48 (30%) to control group 2 (ICUsteps booklet; figure 1). Eighty relatives of the recruited patient participants also agreed to take part.

**Table 3:** ICU patients discharged and recruited per weekday

Recruitment day	Clusters, n (%)	Patient participants recruited, n (%)	Patients discharged, n (%)
Monday	31 (31)	56 (35)	161 (16)
Tuesday	20 (20)	30 (19)	216 (22)
Wednesday	10 (10)	14 (9)	189 (19)
Thursday	16 (16)	18 (11)	226 (23)
Friday	23 (23)	40 (25)	198 (20)
Totals	100 (100)	158 (100)	990 (100)

ICU, intensive care unit.

### Sample demographics

The mean age of patients was 60 (SD 16.04) years (table 4). Participants were predominantly white British/Irish (n=115, 73%) and 82 (52%) were male. Median length of ICU stay was 6 days (range 371). Severity of illness on admission (measured by the APACHE II score) ranged from 4 to 34 (median 17) and on discharge to a ward between 0 and 21 (median 9). Ninety-eight (62%) participants received at least 1 day of level 3 (ICU) care.

**Table 4:** Sample characteristics (patients)

Characteristic	Value	ICUSteps	UCCDIP	Verbal	Total	p Value
Age (years)	Mean±SD	59±15.26	60±15.19	61±17.48	60±16.04	0.72
Ethnicity (white British)	n (%)	34 (71)	40 (78)	41 (69)	115 (73)	0.54
Gender (male)	n (%)	25 (52)	26 (51)	31 (53)	82 (52)	0.99
Medical/Surgical ICU	Medical, n (%)	28 (58)	28 (55)	26 (44)	82 (52)	0.30
Admission type (emergency)	n (%)	38 (79)	40 (78)	44 (75)	122 (77)	0.83
APACHE II score	ICU admission Median (range)	17 (24)	18.0 (30)	16.0 (29)	17.0 (30)	0.41
	ICU discharge Median (range)	8.0 (20)	9.5 (20)	9.0 (21)	9.0 (21)	0.66
Length of stay	ICU days Median (range)	6.0 (62)	7.0 (104)	6.0 (371)	6.0 (371)	0.24
	Hospital days Median (range)	16.0 (132)	21.5 (220)	22.0 (166)	21.0 (221)	0.25
Level 3 critical illness	n (%)	29 (60)	35 (69)	34 (58)	98 (62)	0.58
Total number of participants	n (%)	48 (100)	51 (100)	59 (100)	158 (100)	NA

APACHE, Acute Physiology and Chronic Health Evaluation; ICU, intensive care unit; NA, not available; UCCDIP, user-centred critical care discharge information pack.

For the majority of the sample (n=122, 77%), admission to the ICU was unplanned. Twenty-nine (18%) had experienced previous ICU admissions; 9 of these participants were from the ICUsteps group (n=48, 19%), 10 were from the UCCDIP group (n=51, 20%) and 10 from the ad hoc verbal information group (n=59, 17%). A recorded history of depression with or without anxiety was evident in 14 (9%) of the total patient sample and the presence of delirium while in the ICU was recorded in 11 (7%) participants’ medical notes.

Relatives (n=80) were aged between 18 and 94 years (mean 55 years, SD 14.6), predominantly white British/Irish (n=63, 79%) and female (n=52, 65%). Most were spouses or long-term partners (n=37, 46%) of the recruited patient. A history of anxiety and/or depression was reported by 20 (25%) of the sample.

Patient participants in the UCCDIP sample were more frequently admitted from an in-hospital bed and received more days of level 3 care. They also had higher APACHE II scores on both admission and discharge and stayed in the ICU for longer than participants in either of the other two groups, even when outliers with a ICU stay of >100 days were removed (n=2). None of these differences were statistically significant.

### Participant follow-up

One hundred and one (64%) patient participants provided primary outcome data at time point 1 (5±1 day post-ICU discharge). Fifty-four (34%) were still in hospital and eligible for data collection at time point 2 (28 days or hospital discharge and at least 7 days after their first data collection point). Of these, 38 (70%) provided some data. A total of 48 (60%) patients’ relatives provided at least one set of outcome data.

Twenty-seven (17%) patients and 32 (40%) relatives were lost to any follow-up. By time point 1 (5±1 day), 17 (11%) of the patient sample had already been discharged or transferred from the hospital, and in a further 15 (10%) cases, the patient was either too unwell or unwilling to provide data. In the case of patients’ relatives, the most significant follow-up problem was due to a failure to return data collection forms within the protocol timeframe (n=29, 36%).

#### Hospital anxiety and depression

One week postdischarge (time point 1), median HADS for patients was 7 for anxiety (HADS-A) and 6 for depression (HADS-D). There were no significant differences (p≥0.05) between study groups (table 5). There was, however, a wide range in individual HADS, with almost half the total patient sample (44%) reaching or exceeding the trigger for disorder (≥8). At time point 1, where it was possible to fit a random intercepts model, the estimated ICCs were all low (HADS-A 0.14, HADS-D 0.00, total HADS 0.07).

**Table 5:** HADS (patient sample)

								Mixed model			
		Study group				Kruskal-Wallis		Intervention		Intervention adjusted for day of week	
Outcome	Unit of measurement	ICUsteps	UCCDIP	Verbal	Total	χ^2^ (2df)	p Value	F (dfn,dfd)	p Value	F (dfn,dfd)	p Value
HADS-A: 1*	Median (range)	7.5 (19)	7.0 (17)	6.0 (19)	7.0 (19)	0.98	0.61	0.57 (2,27)	0.57	0.32 (2,27)	0.73
	n	28	31	42	101						
HADS-A: 2†	Median (range)	6.0 (13)	7.0 (18)	5.0 (16)	6.0 (18)	0.08	0.96	0.01‡ (2,35)	0.99	0.00 (2,31)	1.00
	n	8	17	13	38						
HADS-D: 1*	Median (range)	6.5 (18)	6.0 (16)	7.0 (21)	6.0 (21)	0.43	0.80	0.46 (2,24)	0.64	0.41 (2,24)	0.67
	n	28	30	40	98						
HAD-D: 2†	Median (range)	4.5 (16)	6.0 (12)	7.0 (15)	6.5 (16)	0.73	0.70	0.35‡ (2,35)	0.72	0.27‡ (2,31)	0.77
	n	8	17	13	38						
Total HADS: 1*	Median (range)	16.0 (35)	12.5 (32)	14.0 (39)	14.0 (9)	0.44	0.80	0.57 (2,24)	0.57	0.48 (2,24)	0.62
	n	28	30	40	98						
Total HADS: 2†	Median (range)	10.0 (23)	11.0 (27)	12.0 (23)	11.5 (27)	0.41	0.82	0.13‡ (2,35)	0.88	0.10‡ (2,31)	0.90
	n	8	17	13	38						

*5±1 days post-CCU discharge.

†28 days post-CCU discharge or hospital discharge.

‡Model fitted without random intercepts—estimated G matrix not positive definite.

HADS, Hospital Anxiety and Depression Score; HADS-A, HADS for anxiety; HADS-D, HADS for depression; ICU, intensive care unit; UCCDIP, user-centred critical care discharge information pack.

#### Coping and enablement

No significant differences between groups (p≥0.05) for emotion-focused, problem-focused and dysfunctional coping categories or PEI scores were identified at either time point. Over time, the median PEI score for the total patient sample did, however, drop from 12 to 10, indicating that patients felt less enabled the longer they stayed in hospital.

#### Questionnaire findings

Patient participants in the ad hoc verbal information control group reported significantly more chance of worrying a lot (χ^2^=11.16 (df 2), p=0.03) than those in either other study group. However, after using GLIMMIX to adjust for clustering, the effect of the intervention on ‘worry about leaving the Critical Care Unit (CCU)’ was not statistically significant (F (2,39)=0.23, p=0.80). There were no other statistically significant differences in reported feelings or experiences between study groups. However, more participants from the medical as opposed to surgical unit reported that their written information had helped their recovery on the ward, with a result approaching statistical significance (χ^2^=3.69 (df 2), p=0.06).

### Adverse effects

One patient asked to be withdrawn from the trial after data collection point 1 as she felt that completion of the HADS had triggered deterioration in her mental health status. A note was made in her medical file, and she was referred to her primary medical team. There were no other reports of any adverse effects.

### The impact of protocol violations

Twenty-five (16%) patients and 10 (13%) of the patients’ relatives had data collected outside of the time period stated by the protocol for time point 1. At time point 2, the mean time from ICU discharge to data collection was 23±6 days for patients and 25±8.36 days for the relatives.

Including these data produced no change in HADS or PEI outcomes compared with the analysis, which excluded them. At the first follow-up point, however, some significant differences in the scores given for individual questions in the emotion and problem-focused coping categories of the BCOPE were identified. UCCDIP sample data reflect significantly less use of religion (question 12; p=0.01), active coping (question 2; p=0.04) and planning (question 9; p=0.01) strategies, than those participants in either of the other two study groups. Analysis of the composite scores for each coping category (emotion, problem and dysfunctional) also revealed that those in the UCCDIP group used significantly fewer problem-focused coping strategies (H=6.49, p=0.04).

## Discussion

Despite some limited data from previous research, which suggest that written resources may lower levels of patients’ anxiety, depression and symptoms of PTSD,^12^ the health benefits of providing written ICU discharge information remain inconclusive.^5^ Our trial did not find sufficient evidence to determine whether UCCDIP improves patients’ or relatives’ health outcomes or experiences (anxiety, depression, coping, patient enablement) compared with the ICUsteps booklet and/or the delivery of ad hoc verbal information. However, our survey data suggest that those who receive written information may feel less worried about going to a ward than those who receive ad hoc verbal information alone.

The UK Medical Research Council point out that evaluations of draft complex interventions are frequently undermined by numerous practical and methodological problems, and recommend a period of feasibility testing and piloting prior to full scale evaluation.^39^ Using these design principles, data collected during our RCT has identified some important future considerations.

Deciding the optimal time to provide ICU discharge information is an important issue for future practice, particularly considering that recovery rates for physical and emotional recovery may differ.^40^ We gave our intervention to patients immediately prior to their discharge from the ICU. However, the survey data that we collected alongside the trial indicate that many felt unable to engage with the information at this point or during the early days on the ward, and that some of the patients allocated to UCCDIP or the ICUsteps booklet were unaware of having received any written material.^32^ Retention of information is a common problem for ICU patients.^3^ ^4^ Having a family member present when UCCDIP was discussed with the patient may have increased participants’ awareness of and engagement with the intervention. Where possible, such practice is encouraged, particularly if a patient's cognitive function is compromised.

The intention of this trial was to determine the effect of adding UCCDIP into the usual care provided during discharge; thus, after discharge to the ward, no specific instructions were given to ward nurses or other healthcare staff about their role in facilitating its use. Knowledge of the intervention, obtained due to contamination of the allocation concealment after recruitment of the first patient on any day may, however, have influenced staff members’ verbal information delivery, both its quality and quantity. In addition, follow-up personnel, such as discharge coordinators and critical care outreach nurses, have been shown to aid patients’ and relatives’ interaction with written information.^41^ Not providing this support as part of the intervention in this trial may account for some of the problems with engagement that we encountered, particularly for those with English as a second language, poor literacy and/or cognitive impairment. It may also have contributed to the excessive loss to follow-up we experienced, which in turn may have influenced our outcome data. We did not collect data on these participant characteristics and thus are unable to validate these assumptions. These issues are further discussed in Day *et al*.^42^

UCCDIP is a multicomponent intervention, which includes an individualised patient discharge summary written by ICU nurses. Survey data (reported in Bench *et al*^32^) suggest that this element of UCCDIP was of particular value to the patients, relatives and ward nurses who took part in our study. In the protocols for the Scottish RECOVER and RELINQUISH trials,^43^ ^44^ discharge summaries, similar to those used in the present trial, but written by doctors are also included as part of the intervention. In addition, ‘lay summaries’ are now being written by physiotherapists in some parts of the UK (personal communication from Williams N, Edinburgh Critical Care Research Group 6th annual meeting; 26 June 2013). Healthcare professionals’ interest in using patient discharge summaries is also evident by the number of ‘discharge summary training packs’, designed by our project team, being downloaded from the ICUsteps website.^32^ Based on these findings, we recommend that reflective opportunities, such as diaries, are included as part of all individualised rehabilitation programmes.

Our results suggest that medical as opposed to surgical patients may value interventions such as UCCDIP more; perhaps because this group have an increased tendency for psychological problems such as PTSD.^45^ Elective surgical patients may also be better prepared for an ICU admission and their stay is generally expected to be shorter. In our trial, it must be acknowledged, however, that most admissions to the surgical ICU were unplanned. Further, admission to the medical or surgical unit was not always reflective of a patient's condition as beds were used flexibly to meet demand. Despite this limitation, defining subpopulations of critical care (eg, medical vs surgical, ventilated vs non-ventilated) that may benefit most from such an intervention remains an important future consideration, particularly where resources are limited.

### Future research

Evaluating any complex intervention is practically and methodologically difficult.^39^ UCCDIP contains a number of different components, making it difficult to isolate those aspects likely to be most effective. Although findings from the questionnaire survey suggest that the patient discharge summary was considered valuable,^32^ future research is required to examine its effectiveness as a stand-alone intervention.

In this trial, the patients and relatives in all clusters received ad hoc verbal information as part of usual care practice. Ad hoc information delivery can be inconsistent and its quality can differ between healthcare professionals. As in other studies,^13–15^ it was unclear who was delivering the ‘ad hoc’ verbal information during our trial, what was being said or whether it was actually provided at all. Although challenging, attention to qualifying and quantifying these data is a recommendation for further research.

The high prevalence of anxiety and depression experienced by patients and their families in our study suggests that a higher threshold (≥11) on the HADS tool, as used by other researchers^12^ ^46^ ^47^ is required to differentiate the effects of interventions such as UCCDIP on participants. The relationship between emotional status, cognitive appraisal and coping behaviours is complex and individualised. Outcome assessment measures, more closely aligned with the theoretical basis of the intervention (in this case SRT) may, therefore, be better suited to evaluate information interventions in recovering critically ill patients. Development of a validated tool to provide more rigorous data to support the positive views of UCCDIP reported in the locally designed questionnaire survey^32^ is also required.

Our data collection points were specifically chosen to identify any early effects of the intervention on psychological well-being that might influence ongoing recovery. However, the effects of our intervention on patients’ and relatives’ perceived anxiety, depression, coping and enablement may not have been visible during the early stages of recovery.^48^ A longer follow-up period would enable both the early and ongoing impact of different methods of ICU discharge information to be better explored. The common problem of delayed discharge from ICU^49^ should also be considered. Delays can have both positive (physically stronger) and negative effects (increased dependency) on psychological well-being at the point of ICU discharge, and thus may have important implications for evaluating information provision.

It is widely acknowledged that in complex intervention studies such as this, the risk of follow-up bias is high.^39^ Reasons for low follow-up in this study were multifactorial, but key reasons included patients being discharged from hospital earlier than anticipated and relatives failing to return data collection forms. These factors need to be considered in the design of future trials. Including relatives in the evaluation of any critical illness rehabilitation intervention is important, as the family unit often provides substantial support to the patient and close relatives can also be affected by the patient's critical illness.^50^ ^51^ Our experience suggests, however, that once a patient leaves ICU, it is very difficult to maintain communication with relatives and that their commitment to completing study requirements is reduced. Alternate methods of data collection, such as individual telephone interviews, may help reduce the level of attrition we experienced in this study.

In line with best practice recommendations, one of the purposes of this pilot RCT was to inform the power calculation for future work.^52^ Mean HADS-A and HADS-D was 7 (SD 5) in the patient sample. Based on a power of 95% and 0.05% level of significance, to achieve an effect size of 0.4 (difference of 2), a total sample of 286 (143 in each of two groups) would be required for a definitive trial. The different numbers of participants recruited on each weekday (table 1) should also be accounted for. The recruitment rates we observed suggest that this would require a multicentre study to achieve. Given our attrition, it is difficult to judge if such a study would represent value for money although possible benefits for patients include an improved understanding of their critical illness experience, use of more positive coping strategies and improved psychological well-being during the rehabilitation period. Considering the feasibility challenges we experienced and have previously described,^49^ future research could focus on assessing patients’ and relatives’ perceived usefulness of written information resources and the extent to which specific information deemed important is successfully transmitted and retained.

### Limitations

This was a single-centre pilot trial, with a short follow-up period and a high rate of attrition (particularly in the sample of relatives). Only recruiting patients discharged during weekdays and daytime hours may have led to a selection bias, as poorer outcomes are associated with night-time and weekend discharges.^1^ Other potential biases may also have influenced our results. There were some pretest differences between study groups which might have attenuated any potential benefits. HADS were skewed and the sample was small at time point 2. This should be borne in mind when interpreting the statistical model; however, the results were consistent whichever approach was used. There was also a failure to maintain allocation concealment after recruitment of the first patient in each cluster and a possible Hawthorne effect, where staff may have provided verbal information differently from normal because they were aware of the nature of the study.

Results must therefore be viewed with caution and may not be generalisable to the wider critical care population. The study has, however, provided important data, which can inform future trials evaluating interventions like UCCDIP, enabling processes to be streamlined and a sample size based on a more accurate power calculation to be used.^52–54^

## Conclusion

This single-centre pragmatic pilot RCT used cluster randomisation to undertake an initial evaluation of UCCDIP, a discharge information pack designed by the project team. We were unable to prove the effectiveness of UCCDIP, supporting the view that information giving to those recovering from critical illness is a complex intervention. This research has, however, provided important preliminary data regarding how, when and for whom an intervention based on the principles of UCCDIP could be most effective and what it would look like.

To increase the likelihood of similar interventions improving health outcomes, key considerations for future work are: (1) medical as opposed to surgical critical care patients may be more likely to benefit from such interventions; (2) after discharge to the ward, patients need further input and support to help them engage fully with written information resources; (3) data collection time points should reflect the potential effects on both early and later recovery; and (4) outcome measures more sensitive to the effects of UCCDIP should be used for future evaluations.

## References

[1] National Institute for Health and Clinical Excellence (NICE). Rehabilitation after critical illness. 2009 http://www.nice.org.uk. (accessed Oct 2014). 20704055

[2] Harvey MA, Davidson J Long term consequences of critical illness: a new opportunity for high impact critical care nurses. Crit Care Nurse 2011;31:12–15. 10.4037/ccn2011597 21965379

[3] Bench S, Day T The user experience of critical care discharge; a meta-synthesis of qualitative research. Int J Nurs Stud 2010;47:487–99. 10.1016/j.ijnurstu.2009.11.013 20004396

[4] Bench S, Day T, Griffiths P Involving users in the development of effective critical care discharge information: a focus group study. Am J Crit Care 2011;20:443–52. 10.4037/ajcc2011829 22045141

[5] Bench S, Day T, Griffiths P Effectiveness of critical care discharge information in supporting early recovery from critical illness. Crit Care Nurse 2013;33:41–52. 10.4037/ccn2013134 23727851

[6] Department of Health (DH). Making Partnership Work for Patients, Carers and Service users: a strategic agreement between the Department of Health, the NHS and the voluntary and community sector. 2004 http://webarchive.nationalarchives.gov.uk/+/www.dh.gov.uk/en/Publicationsandstatistics/Publications/PublicationsPolicyAndGuidance/DH_4089515. (accessed Oct 2014).

[7] Department of Health (DH). Self care; a real choice. Self care support—a practical option. London: Crown copyright, 2005.

[8] Coulter A, Ellis J Patient Focused Interventions; a review of the evidence. London: The Health Foundation, 2006.

[9] Department of Health (DH). Our health, our care, our say; a new direction for community services. London: Crown copyright, 2006.

[10] National Institute for Health and Clinical Excellence (NICE). Acutely Ill patients in hospital; recognition and response to acute illness in adults in hospital. 2007 http://www.nice.org.uk. (accessed Oct 2014). 21204323

[11] Cutler L, Hayter M, Ryan T A critical review and synthesis of qualitative research on patient experiences of critical illness. Intensive Care Nurs 2013;29:147–57. 10.1016/j.iccn.2012.12.001 23312486

[12] Jones C, Skirrow P, Griffith RD Rehabilitation after critical illness: a randomized controlled trial. Crit Care Med 2003;31:2456–61. 10.1097/01.CCM.0000089938.56725.33 14530751

[13] Mitchell M, Courtney M Reducing family members’ anxiety and uncertainty in illness around transfer from intensive care: an intervention study. Intensive Care Nurs 2004;20:223–31. 10.1016/j.iccn.2004.05.008 15288876

[14] Paul F, Hendry C, Cabrelli L Meeting patient and relatives’ information needs upon transfer from an intensive care unit: the development and evaluation of an information booklet. J Clin Nurs 2004;13:396–405. 10.1046/j.1365-2702.2003.00876.x 15009342

[15] Kitchens J Effectiveness of an intensive care unit transfer brochure on family members’ associated knowledge and anxiety. Clin Nurse Spec 2009;23:99 10.1097/01.NUR.0000325412.53976.d9

[16] National Institute for Health Research (NIHR) Evaluation, Trials and Studies Coordinating Centre (NETSCC). Research methods; feasibility and pilot studies. 2013 http://www.netscc.ac.uk/glossary/#glos8. (accessed Octr 2014).

[17] INVOLVE. Briefing Notes for Researchers: public involvement in NHS, public health and social care research. 2012 http://www.invo.org.uk/wp-content/uploads/2012/04/INVOLVEBriefingNotesApr2012.pdf. (accessed October 2014).

[18] Morrow E, Boaz A, Brearley S Handbook of service user involvement in nursing and healthcare research. Chichester: Wiley-Blackwell, 2012.

[19] Department of Health (DH). Comprehensive critical care; a review of adult critical care services. London: Crown copyright, 2000.

[20] Her Majesty's Stationary Office (HMSO). Mental Capacity Act. 2005 http://www.legislation.gov.uk/ukpga/2005/9/contents. (accessed Oct 2014).

[21] Leventhal H, Meyer D, Nerenz D The common sense representation of illness danger. In: Rachman S, eds. Contributions to medical psychology. Vol 2 New York: Pergamom press, 1980:7–30.

[22] Johnson J, Lauver D Alternative explanations of coping with stressful experiences associated with physical illness. Adv Nurs Sci 1989;11:39–52. 10.1097/00012272-198901000-00008 2493768

[23] Hagger M, Orbell S A meta-analytic review of the common sense model of illness representations. Psychol Health 2003;18:141–84. 10.1080/088704403100081321

[24] Bench S, Day T, Griffiths P Developing user centred critical care discharge information to support early critical illness rehabilitation using the Medical Research Council's complex interventions framework. Intensive Care Nurs 2012;28:123–31. 10.1016/j.iccn.2012.02.002 22386848

[25] Fogg L, Gross D Threats to validity in randomized clinical trials. Res Nurs Health 2000;23:79–87. 10.1002/(SICI)1098-240X(200002)23:1<79::AID-NUR9>3.0.CO;2-R 10686575

[26] Finiss DG, Kaptchuk TJ, Miller F Biological, clinical and ethical advances of placebo effects. Lancet 2010;375:686–95. 10.1016/S0140-6736(09)61706-2 20171404 PMC2832199

[27] ICUsteps: Intensive care; a guide for patients and relatives. 2010 http://www.icusteps.org. (accessed Oct 2014).

[28] Zigmond A, Snaith R The hospital anxiety and depression scale. Acta Psychiatr Scand 1983;67:361–70. 10.1111/j.1600-0447.1983.tb09716.x 6880820

[29] Bjelland I, Dahl AA, Haug TT The validity of the hospital anxiety and depression scale; an updated literature review. J Psychosom Res 2002;52:62–77. 10.1016/S0022-3999(01)00296-3 11832252

[30] Carver C You want to measure coping but your protocol's too long: consider the Brief COPE. Int J Behav Med 1997;4:92–100. 10.1207/s15327558ijbm0401_6 16250744

[31] Howie JG, Heaney DJ, Maxwell M A comparison of a Patient Enablement Instrument (PEI) against two established satisfaction scales as an outcome measure of primary care consultations. Fam Pract 1998;15:165–71. 10.1093/fampra/15.2.165 9613486

[32] Bench SD, Heelas K, White C Providing critical care patients with a personalised discharge summary: a questionnaire survey and retrospective analysis exploring feasibility and effectiveness. Intensive Care Nurs 2014;30:69–76. 10.1016/j.iccn.2013.08.007 24211048

[33] Knaus WA, Draper EA, Wagner DP APACHE II: a severity of disease classification system. Crit Care Med 1985;13:818–29. 10.1097/00003246-198510000-00009 3928249

[34] Gammon J, Mulholland CW Effect of preparatory information prior to elective total hip replacement on psychological coping outcomes. J Adv Nurs 2006;24:303–8. 10.1046/j.1365-2648.1996.17911.x 8858434

[35] Medical Research Council (MRC). Cluster randomised trials: methodological and ethical considerations. 2002 http://www.mrc.ac.uk/Utilities/Documentrecord/index.htm?d=MRC002406-. (accessed Oct 2014).

[36] Hayes R, Moulton L Cluster randomised trials, Interdisciplinary statistics series. Florida: Chapman & Hall, 2009.

[37] White I, Carpenter J, Horton N Including all individuals is not enough: lessons for intention-to-treat analysis. PLoS Clin Trials 2012;9:396–407. 10.1177/1740774512450098 PMC3428470 22752633

[38] CONsolidated Standards of Reporting Trials (CONSORT). Baseline data. 2010 guideline. Section 15. http://www.consort-statement.org/checklists/view/32-consort/510-baseline-data. (accessed May 2015). 10.1016/j.jclinepi.2010.01.002 20346626

[39] Medical Research Council (MRC). Developing and evaluating complex interventions: new guidance. 2008 http://www.mrc.ac.uk/Utilities/Documentrecord/index.htm?d=MRC004871. (accessed Oct 2014).

[40] Desai S, Law T, Needham D Long-term complications of critical care. Crit Care Med 2011;39:371–9. 10.1097/CCM.0b013e3181fd66e5 20959786

[41] Chaboyer W, Thalib L, Alcorn K The effect of an ICU liaison nurse on patients and family's anxiety prior to transfer to the ward: an intervention study. Intensive Care Nurs 2007;23:362–9. 10.1016/j.iccn.2007.04.005 17681470

[42] Day T, Bench S. Griffiths P The role of pilot testing for a randomised control trial of a complex intervention in critical care. J Res Nurs 2015;20:167–78.

[43] Ramsay P, Huby G, Rattray J A longitudinal qualitative exploration of healthcare and informal support needs among survivors of critical illness: the RELINQUISH protocol. BMJ Open 2012;2:pii e001507 10.1136/bmjopen-2012-001507 PMC3400070 22802422

[44] Walsh TS, Salisbury LG, Boyd J A randomised controlled trial evaluating a rehabilitation complex intervention for patients following intensive care discharge: the RECOVER study. BMJ Open 2012;2:pii e001475 10.1136/bmjopen-2012-001475 PMC3391367 22761291

[45] Jackson J, Hart R, Gordon S, et al Post-traumatic stress disorder and post-traumatic stress symptoms following critical illness in medical intensive care unit patients: assessing the magnitude of the problem. Crit Care 2007;11:R27 10.1186/cc5707 17316451 PMC2151890

[46] Knowles R, Tarrier N Evaluation of the effect of prospective patient diaries on emotional well-being in intensive care unit survivors: a randomized controlled trial. Crit Care Med 2009;37:184–91. 10.1097/CCM.0b013e31819287f7 19050634

[47] Garrouste-Orgeas M, Coquet I, Périer A Impact of an intensive care unit diary on psychological distress in patients and relatives. Crit Care Med 2012;40:2033–40. 10.1097/CCM.0b013e31824e1b43 22584757

[48] Eddleston J, White P, Guthrie E Survival, morbidity, and quality of life after discharge from intensive care. Crit Care Med 2000;28:2293–9. 10.1097/00003246-200007000-00018 10921555

[49] Mellinghoff J, Rhodes A, Grands M Factors and consequences associated with a delay in the discharge process of patients from an adult critical care unit. Crit Care 2011;15:463 10.1186/cc9883

[50] Davidson JE, Jones C, Bienvenue OJ Family response to critical illness: post intensive care syndrome-family. Crit Care Med 2012;40:618–24. 10.1097/CCM.0b013e318236ebf9 22080636

[51] Griffiths J, Hatch R, Bishop J An exploration of social and economic outcome and associated health-related quality of life after critical illness in general intensive care unit survivors: a 12 month follow up study. Crit Care 2013;17:R100 10.1186/cc12745 23714692 PMC3706775

[52] Lancaster G, Dodd S, Williamson P Design and analysis of pilot studies: recommendations for good practice. J Eval Clin Pract 2004;10:307–12. 10.1111/j.2002.384.doc.x 15189396

[53] Arnold D, Burns K, Adhikari N The design and interpretation of pilot trials in clinical research in critical care. Crit Care Med 2009;37:S69–74. 10.1097/CCM.0b013e3181920e33 19104228

[54] Arain M, Campbell MJ, Cooper CL What is a pilot or feasibility study? A review of current practice and editorial policy. BMC Med Res Methodol 2010;10:67 10.1186/1471-2288-10-1 20637084 PMC2912920

